# Prevalence of reactive allergens in contact patch testing studies using the Brazilian standard battery: a systematic review^☆^

**DOI:** 10.1016/j.abd.2025.501126

**Published:** 2025-06-23

**Authors:** Ana Laura Andrade Bueno, Nathalia Hoffmann Guarda Aguzzoli, Renan Rangel Bonamigo

**Affiliations:** a Graduate Program in Pathology, Universidade Federal de Ciências da Saúde de Porto Alegre, Porto Alegre, RS, Brazil; b Internal Medicine Department, Dermatology Service, Universidade Federal do Rio Grande do Sul, Porto Alegre, RS, Brazil

**Keywords:** Brazil, Dermatitis, contact, Patch test

## Abstract

**Background:**

Contact dermatitis is a common dermatosis in dermatology. The patch test is the gold standard for diagnosis, using a panel of common allergens and irritants. In Brazil, a standardized battery of 30 substances is used. However, comprehensive epidemiological data on contact dermatitis are limited, and no studies have compiled and compared nationwide results.

**Objectives:**

This study aimed to assess the positivity rate of patch tests using the Brazilian Standard Battery in suspected contact dermatitis cases from 2000 to 2022. We also evaluated associations between reactive substances and clinical and demographic variables and examined the variation in reactivity frequency to allergenic substances over the study period.

**Methods:**

A systematic literature review was conducted using PubMed, Scielo, and LILACS. We evaluated the prevalence of positive tests for each substance, examined associations between variables such as gender, region, and age, and assessed variations in patch test positivity over time.

**Results:**

Initially, 55 articles were identified, and 10 were included in the review. Nickel sulfate was the most frequently positive allergen, while triclosan, P-tertiary buthyphenol, and anthraquinone were the least prevalent. Test positivity increased every 5-years for kathon CG, neomycin, and nickel sulfate, while it decreased for quaternion 15 and thimerosal.

**Study limitations:**

The results were limited by the small number of articles included in the review.

**Conclusions:**

This pioneering study in Brazil provides valuable insights for dermatological allergy practitioners into the prevalence of patch test positivity for each substance in the Brazilian Standard Battery, aiding in informed decision-making and patient management.

## Introduction

Contact dermatitis (CD) is a prevalent inflammatory skin condition frequently encountered in dermatological practice. Its clinical presentation is characterized by erythematous, desquamative, and pruritic vesicles, papules, or plaques, often accompanied by lichenification, leading to a significant impact on patients' quality of life⁠.^1^ The CD comprises a significant proportion of occupational dermatoses and carries substantial socioeconomic implications, particularly in industrialized nations.^2^⁠⁠

CD can be classified into two main types: Irritant contact dermatitis (ICD) and allergic contact dermatitis (ACD). ICD arises from the direct toxic and pro-inflammatory effects of certain substances, while ACD is a delayed-type hypersensitivity reaction mediated by hapten-specific T-cells⁠⁠.^1^

While the diagnosis of CD primarily relies on clinical evaluation, patch testing (PT) serves as the gold standard complementary diagnostic tool for confirming this condition. PT aims to reproduce the elicitation phase of ACD by controlled exposure to suspected agents, thereby confirming the diagnosis and determining the etiology of ACD. The PT series typically consists of a standard battery of common allergens and irritants, including fragrances, preservatives, metals, rubber, and other chemicals to which patients are commonly exposed and may be associated with their clinical presentation.^2^, ^3^

In Brazil, the Brazilian Group for the Study of Contact Dermatitis (GBEDC) developed a standardized battery in 2000, approved by the Brazilian Society of Dermatology (SBD), comprising 30 substances. However, there is a scarcity of Brazilian etiological and epidemiological data on ACD across different regions that could provide population representativeness. While several studies have been published by prominent Brazilian dermatological centers, they have been conducted in isolation, and no comprehensive analyses or comparisons of results have been published at the national level⁠⁠.^2^, ^3^

It is well-established that the population profile and exposure to various substances can influence PT results, which are also known to evolve over time. Therefore, to address these gaps in knowledge, a systematic review was undertaken with the objective of collating and synthesizing information regarding PT results obtained using the Brazilian standard battery over the past 22-years.

## Methods

### Study design

This study presents a systematic review (SR) conducted between 2000 and 2022, with the aim of identifying relevant articles published in English, Portuguese, or Spanish. It was conducted in accordance with the Preferred Reporting Items for Systematic Reviews and Meta-Analyses (PRISMA).^4^

A comprehensive search was performed by two screeners who independently searched scientific databases known for housing Brazilian studies, namely PubMed, LiLACS, and SciELO. The primary objective was to gather a comprehensive body of literature to provide valuable insights into the medical field. The plan to establish data on the subject between 2000 and 2022 is related to the new research, being carried out by the Study of Contact Dermatitis and the Brazilian Society of Dermatology, which began in 2023. Articles published after 2022 will be commented on and not included in the analysis.

### Data extraction and quality assessment

In the initial stage, a comprehensive assessment was conducted by two researchers to identify and remove duplicate articles obtained from the search process. Subsequently, a meticulous examination of the titles and abstracts of the retrieved records was performed, resulting in the exclusion of studies that did not meet the predetermined eligibility criteria.

In the subsequent stage, the full texts of the remaining studies were thoroughly evaluated by the authors, employing strict inclusion and exclusion criteria. This critical appraisal enabled the identification of studies that met the predefined criteria and were deemed eligible for further analysis.

#### Selection criteria

In order to ensure the inclusion of relevant articles, the following selection criteria were applied:

• Original articles;
• Articles with full texts available;
• Studies that performed contact tests with the Brazilian Standard Battery (Brazilian Contact Dermatitis Study Group ‒ GBEDC);
• Studies with full results of all substances tested in patch tests;
• Studies conducted in Brazil;
• Studies that evaluated the population of patients diagnosed or suspected of having contact dermatitis;
• Studies published between 2000‒2022.

#### Exclusion criteria

The following criteria were employed to exclude articles from consideration:

• Ongoing clinical trials without published results and case reports;
• Articles with specific subpopulations;
• Studies that performed contact tests with batteries other than the Brazilian Standard Battery (Brazilian Contact Dermatitis Study Group ‒ GBEDC);
• Studies are not conducted in Brazil.

Quality assessment was performed using the Joanna Briggs Institute Critical Appraisal Tool.

The checklist for cross-sectional studies and the checklist for cohort studies were selected and applied, according to the study design^5^:

• Checklist for cross-sectional studies, in which studies were scored on 8 items. Each item was evaluated using the options: yes, no, unclear, not applicable. The authors considered articles to be of good quality if they received a “yes” response for all evaluated items; of medium quality if they received a “yes” response for 5 or more items; and of low quality if they received a “yes” response for 4 or fewer items.
• Checklist for cohort studies, in which studies were scored on 11 items. Each item was evaluated using the options: yes, no, unclear, not applicable. The authors considered articles to be of good quality if they received a “yes” response for all evaluated items; of medium quality if they received a “yes” response for 6 or more items; and of low quality if they received a “yes” response for 5 or fewer items.

### Search strategy

A comprehensive search was performed by two screeners who independently searched in scientific databases known for housing Brazilian studies, namely PubMed, LiLACS, and SciELO. The primary objective was to gather a comprehensive body of literature to provide valuable insights into the medical field.

The selection of search terms was guided by their relevance to the specific medical condition under investigation, encompassing both general and specific keywords. The search was conducted on October 10, 2022, and the search strategy utilized on each platform will be detailed below:

• PubMed: (“patch tests”[MeSH Terms] OR (“patch”[All Fields] AND “tests”[All Fields]) OR “patch tests”[All Fields] OR (“patch”[All Fields] AND “test”[All Fields]) OR “patch test”[All Fields]) AND (“Brazil”[MeSH Terms] OR “Brazil”[All Fields] OR “Brazil s”[All Fields] OR “Brazils” [All Fields]);
• LiLACS: (“teste de contato” or “patch test”) (Brasil or Brazil);
• SciELO: (patch test) OR (teste de contato) AND (Brazil).

To enhance the comprehensiveness of the search strategy and yield a robust dataset for analysis, reference lists of the included studies were reviewed to identify additional studies that may have been missed through the initial search process. This comprehensive approach ensured the inclusion of relevant studies within the Brazilian context.

### Variables

The study examined the following variables:

• Reactivity of the 30 substances comprising the Brazilian Standard Battery;
• Gender (Sex);
• Age;
• Geographical region of Brazil.

### Statistical analysis

To determine the prevalence of positive substance tests, a weighted summary meta-analysis mean was utilized, considering the sample size of each study as a weighting factor.

For the association tests, eight composite and simple linear regressions of meta-analysis were tested to select the best model. The outcome measure used was the percentage of positive contact tests, with combinations of the study's percentage of female participants, categorized study region (with the Southeast region as the reference and the South region as the comparison), mean age of study participants (with and without a constant). To assess changes in contact test positivity rates over time, the mean value of each substance within each time interval and their corresponding coefficients were calculated using simple linear regression, considering time as a continuous variable.

## Results

**Figure 1:**
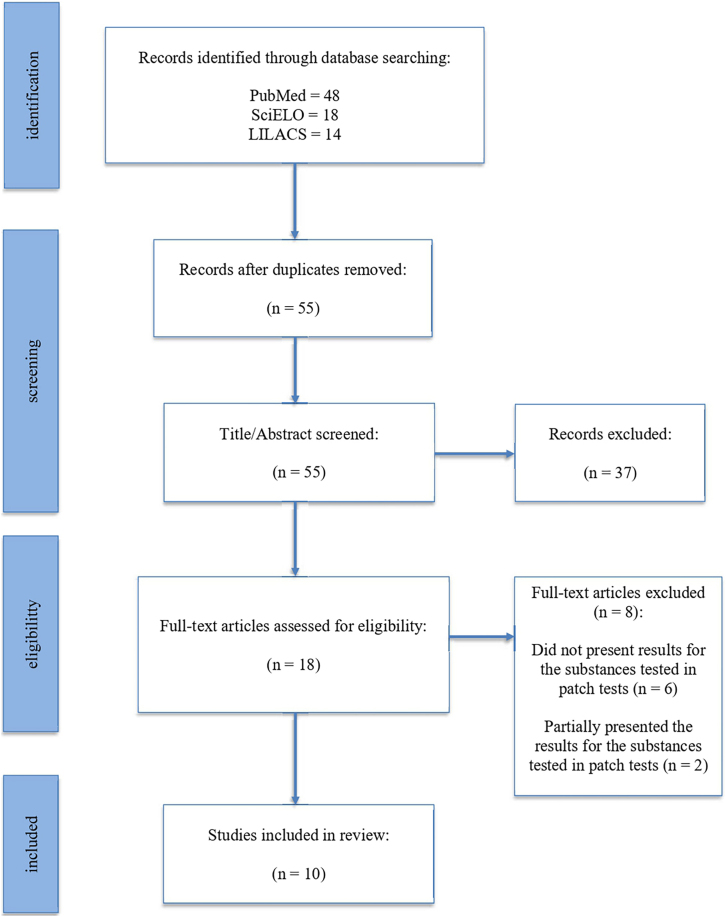
Search strategy results.

Through the initial search, a total of 81 articles deemed potentially relevant were identified. Following the removal of duplicates, 55 articles remained for further evaluation. Subsequently, 37 articles were excluded based on title and abstract screening. The full texts of the remaining 18 articles were thoroughly assessed for eligibility. Ultimately, 10 studies met the predefined inclusion criteria and were included in this systematic review, as depicted in Fig. 1. Table 1 provides a summary of the selected studies and their respective characteristics.

**Table 1:** Summary of the selected studies and their respective characteristics.

First Author/Year	Study design	City/Region	Evaluated period	Sample Size	Mean age	Sex (%)	Results: positive substances (%)
Oppermann et al. 2021	Cohort (prospective)	Porto Alegre South	2018 to 2019	77	41.3	F: 77.9	Nickel sulfate 38
					*Unrepresented Variability	M: 22.1	Kathon CG 15
					*Minimum‒maximum: 3‒75 years		Perfume mix 14
							Paraphenylenediamine 9
							Neomycin, Thimerosal 8
							Cobalt chloride 7
							PPD mix, Carba mix 6
							Balsam-of-Peru, Potassium dichromate, P-tertiary Buthylphenol 5
							Thiuram mix, Colophony 4
							Etilenodiamine 3
							Propylene Glycol, Benzocaine, Epoxi-resine 2
							Anthraquinone, Hydroquinone, Mercapto mix, Quaternium-15, Quinine mix, Nitrofurazone, Paragon mix, Terebentine 1
Rodrigues et al. 2015	Cross-sectional	Belo Horizonte Southest	2003 to 2010	125	14.3 ± 3.8	F: 76.8	Nickel sulfate 46
						M: 23.2	Thimerosal 23
							Neomycin 8
							Cobalt chloride, Perfume mix 5
							Formaldehyde 4
							Potassium dichromate, Etilenodiamine 3
							PPD mix, Balsam-of-Peru, Quinine mix
							Anthraquinone, Kathon CG, Hydroquinone, Mercapto mix 2
							Quaternium-15, Promethazine 1
							Colophony, Carba mix
Duarte et al. 2013	Cohort (restrospective)	São Paulo Southest	2006 to 2011	618	*Unrepresented variability	F: 68.4	Nickel sulfate 174
					*Predominant age group: 30‒49 years	M: 31.6	Thimerosal 99
							Potassium dichromate 69
							Cobalt chloride 65
							Perfume mix 54
							Neomycin, PPD mix 45
							Paraphenylenediamine 43
							Carba mix 41
							Thiuram mix 38
							Etilenodiamine 27
							Promethazine 21
							Balsam-of-Peru, Formaldehyde 20
							Mercapto mix 19
							Hydroquinone, Quaternium 15
							Colophony 17
							Quinine mix, Lanoline 16
							Kathon CG, Benzocaine 15
							Paragon mix, Nitrofurazone 14
							Terebentine 11
							Propylene Glycol 10
							Epoxi-resine 9
							Irgasan, P-tertiary Buthylphenol 5
							Anthraquinone 4
Rodrigues et al. 2012	Cohort (restrospective)	Belo Horizonte Southest	2003 to 2010	1406	42 ± 16	F: 69.7	Nickel sulfate 442
						M: 30.3	Thimerosal 207
							Potassium dichromate 114
							Paraphenylenediamine 99
							Cobalt chloride 97
							Perfume mix 94
							Neomycin 88
							Balsam-of-Peru, Formaldehyde 72
							Etilenodiamine 67
							PPD mix 60
							Carba mix 40
							Thiuram mix 37
							Colophony 30
							Hydroquinone 25
							Paragon mix 22
							Kathon CG 20
							Quaternium 15, Nitrofurazone 19
							Propylene Glycol 10
							Benzocaine 16
							Epoxi-resine 12
							Mercapto mix 14
							Quinine mix 14
							Terebentine, triclosan 11
							Promethazine 17
							Anthraquinone 7
							Lanoline, P-tertiary Buthylphenol 6
Silva et al. 2020	Cohort (restrospective)	São Paulo Southest	2015 to 2017	267	43 ± 16	F:72.9	Nickel sulfate 108
						M:27.1	Cobalt chloride 45
							Neomycin 35
							Potassium dichromate 34
							Kathon CG 29
							Thimerosal 28
							Formaldehyde 25
							Paraphenylenediamine 18
							Perfume mix 16
							Colophony 11
							Balsam-of-Peru 9
							Triclosan 8
							Carba mix, Thiuram mix 6
							Etilenodiamine, Paragon mix 5
							Quaternium 15, Quinine mix, 4
							Nitrofurazone, Epoxi-resine
							Lanoline, Mercapto mix 3
							Promethazine, Hydroquinone, 2
							Benzocaine, PPD mix
							Terebentine, P-tertiary Buthylphenol 1
GBEDC et al. 2000	Cohort (prospective)	São Paulo Southest	1995 to 1996	967	37.1 ± 15	F: 62.5	Nickel sulfate 243
						M: 37.5	Thimerosal 158
							Quaternium 15 109
							Cobalt chloride 106
							Paraphenylenediamine 84
							Perfume mix 81
							Potassium dichromate 78
							Quinine mix 58
							Thiuram mix 53
							Neomycin 42
							Formaldehyde 37
							Carba mix, Mercapto mix, Nitrofurazone 36
							Balsam-of-Peru 31
							Etilenodiamine 30
							Paragon mix 26
							Colophony 25
							Epoxi-resine, PPD mix, Promethazine 17
							Kathon CG 21
							Terebentine 14
							Hydroquinone 13
							Lanoline 11
							triclosan, P-tertiary Buthylphenol 7
							Anthraquinone 5
Correa et al. 2018	Cross-sectional	Santa Catarina South	2004 to 2013	539	38.8	F: 75,9	Nickel sulfate 196
					*Unrepresented variability	M: 24.1	Cobalt chloride 95
							Thimerosal 81
							Paraphenylenediamine 44
							Potassium dichromate 43
							Kathon CG 42
							Carba mix 39
							Formaldehyde 38
							Perfume mix 37
							PPD mix 35
							Promethazine 32
							Neomycin 28
							Colophony 24
							Terebentine 22
							Etilenodiamine 18
							Hydroquinone 15
							Epoxi-resine 14
							Nitrofurazone, Paragon mix 12
							Propylene Glycol, P-tertiary Buthylphenol 11
							Balsam-of-Peru, Thiuram mix 10
							Irgasal 9
							Anthraquinone, Quinine mix 7
							Quaternium 15 6
							Benzocaine 5
							Mercapto mix 4
							Lanoline 4
Fernandes et al. 2007	Cohort (prospective)	São Paulo Southest	2001 to 2002	65	45.1	F: 90.7	Nickel sulfate 24
					*Unrepresented variability	M: 9.23	Thimerosal 13
							Neomycin 6
							Formaldehyde 5
							Cobalt chloride 3
							Mercapto mix, Lanolin, Perfume mix, PPD mix, Paraphenylenediamin 2
							Balsam-of-Peru, Thiuram mix
							P-tertiary Buthylphenol, Etilenodiamine, Epoxi-resine 1
Duarte et al. 2007	Cohort (prospective)	São Paulo Southest	1998 to 2003	506	*Unrepresented mean and variability	F: 64	Nickel sulfate 180
						M:36	Cobalt chloride 85
							Thimerosal 64
							Potassium dichromate 61
							Perfume mix 58
							Paraphenylenediamine, Neomycin, Thiuram mix 47
							Quaternium-15 45
							Carba mix, Nitrofurazone 34
							Etilenodiamine 27
							Quinine mix 23
							PPD mix 22
							Balsam-of-Peru, Epoxi-resine 21
							Paragon mix, Promethazine 20
							Colophony 18
							Lanoline 12
							Hydroquinone 10
							Benzocaine 9
							Formaldehyde 7
							Propylene Glycol 6
							Anthraquinone 3
							P-tertiary Buthylphenol triclosan 2
Artus et al.	Cross-sectional	Porto Alegre/South	2007 to 2010	133	42 ± 15	F: 69.2	Nickel sulfate
						M: 68.7	Thimerosal 45
							Paraphenylenediamine 25
							Neomycin 22
							Cobalt chloride 19
							PPD mix 17
							Potassium dichromate 16
							Carba mix 14
							Perfume mix, Thiuram mix 12
							Kathon CG, Mercapto mix, Paragon mix, 10
							Promethazine, Hydroquinone 8
							Balsam-of-Peru, Colophony, Epoxi-resine, Terebentine 7
							Etilenodiamine, Formaldehyde, triclosan, 6
							Anthraquinone, Nitrofurazone 5
							Mercapto mix 4
							Lanoline, Quaternium 15, Quinine mix, Propylene Glycol, Benzocaine,
							P-tertiary Buthylphenol 3

Considering the criteria of the tool used for quality assessment, the Joanna Briggs Institute Critical Appraisal Tool, the authors found 7 articles of medium quality,^2^, ^3^, ^6^, ^7^, ^8^, ^11^, ^13^ 3 considered to be of good quality,^9^, ^10^, ^12^ and none of the 10 articles pre-selected based on the inclusion and exclusion criteria were considered to be of low quality, with no indication for exclusion from the systematic review.

The total number of patients subjected to patch testing, considering all selected studies, was 4703. In all 10 studies, the tests were performed on patients with suspected contact dermatitis. The studies were conducted in four different states in Brazil, but only in two regions, in the South and Southeast. ⁠⁠In the South region, there was one study in the state of Santa Catarina and two studies in the state of Rio Grande do Sul.^2^, ^6^, ^7^ In the Southeast region, there were five studies in the state of São Paulo^8^, ^9^, ^10^, ^11^, ^12^ and two studies in the state of Minas Gerais.^3^, ^13^⁠⁠ Regarding gender, the majority of the population included in the studies was female in all 10 studies, as shown in detail in Table 1.

As for the sites of dermatitis, the cephalic segment (face, head, and neck) was the most affected in all studies, except in Rodrigues et al.,^13^ where it ranked as the second most common after the hands. In Fernandes et al.^9^⁠⁠ the most common sites were eyelids (12.30%) and face (9.2%); in Silva et al.,^12^⁠⁠ the cephalic segment including the head, face, and neck accounted for 44.79% of cases; in Duarte et al.,^11^ the cephalic segment accounted for 45.3% of cases; in Rodrigues et al.⁠⁠,^3^ the face was affected in 24.8% of cases; in Artus et al.,^6^ the head and neck were affected in 48.9% of cases; and in Duarte et al.,^8^ the cephalic segment (head and neck) was the most frequently affected (81 cases, 29%). In Rodrigues et al.,^13^ the hands were the most affected site (44.7%), and the studies by Opperman et al.,^7^ GBEDC10⁠⁠, and Correa et al.^2^ did not provide this information.

### Technique

All studies utilized the Brazilian standard battery, composed of 30 substances recommended by the Brazilian Group of Studies in Contact Dermatitis, originally produced by FDA-Allergenic/Immunothec (RJ, Brazil).

All studies followed the criteria of the International Contact Dermatitis Research Group (ICDRG) for application and interpretation techniques. Readings were performed at 48 and 96 hours, according to the established criteria. Positive tests at 96 hours related to current and past history of contact dermatitis were considered relevant.^10^⁠

### Frequency of most common allergens

Among adults with suspected Allergic Contact Dermatitis (ACD), the most frequently positive allergen in patch tests was nickel sulfate, with an average prevalence of 31.8%, as shown in Table 2. Individually, it was also the test with the highest positivity in the 10 studies that examined this population, with percentages ranging from 28.1%^11^⁠ to 56.2%^12^⁠⁠ of the tested patients in each study.

**Table 2:** Prevalence of positive patch test reactions in Brazil, by substance, between 2000‒2022 (n = 4.703).

					Confidence Interval (90%)	
Substance	Positive tests*	Mean (%)	Standard Deviation	Standard Error	Lower Limit (%)	Upper Limit (%)
Nickel sulfate	1.496	31.8	25.3	8.00	17.2	46.5
Thimerosal	727	15.5	3.2	1.02	13.6	17.3
Cobalt chloride	504	10.7	14.3	4.51	2.4	19.0
Potassium dichromate	421	9.0	5.4	1.71	5.8	12.1
Para-phenylenediamine	368	7.8	5.1	1.61	4.9	10.8
Perfume mix	362	7.7	5.4	1.71	4.6	10.8
Neomycin	335	7.1	6.1	1.92	3.6	10.6
Carba mix	219	4.7	5.0	1.58	1.8	7.6
Formaldehyde	214	4.6	4.1	1.29	2.2	6.9
Thiuram mix	206	4.4	6.1	1.92	0.9	7.9
Quaternium 15	204	4.3	17.7	5.60	0.0	14.6
PPD mix	203	4.3	5.4	1.71	1.2	7.5
Ethylenediamine	187	4.0	1.0	0.32	3.4	4.6
Balsam of Peru	178	3.8	1.5	0.47	2.9	4.6
Kathon CG	151	3.2	12.9	4.10	0.0	10.7
Colophony	137	2.9	1.1	0.34	2.3	3.5
Nitrofurazone	125	2.7	3.0	0.94	0.9	4.4
Quinoline mix	122	2.6	4.1	1.29	0.2	5.0
Promethazine	118	2.5	3.1	0.98	0.7	4.3
Paraben mix	113	2.4	1.2	0.38	1.7	3.1
Hydroquinone	94	2.0	1.0	0.31	1.4	2.6
Epoxy resin	87	1.8	1.4	0.44	1.0	2.7
Mercapto mix	85	1.8	1.8	0.58	0.8	2.9
Turpentine	67	1.4	1.7	0.55	0.4	2.4
Benzocaine	57	1.2	0.5	0.15	0.9	1.5
Lanolin	56	1.2	0.8	0.24	0.7	1.6
Propylene glycol	50	1.1	0.4	0.12	0.8	1.3
Triclosan	47	1.0	0.8	0.25	0.5	1.5
p-Tertiary butylphenol	41	0.9	0.9	0.27	0.4	1.4
Anthraquinone	33	0.7	0.4	0.12	0.5	0.9

* Unweighted N.

The second most frequently positive allergen was thimerosal and cobalt chloride, with an average prevalence of 15.5% and 10.7%, respectively. Both substances were among the top 5 most frequently positive allergens in all articles.

Considering the metal series, in addition to the high prevalence of nickel sulfate and cobalt chloride, potassium dichromate presented an average prevalence of 9%.

Among the seven most frequent substances in the included studies, the authors should also mention fragrance mix (7.7%), paraphenylenediamine (7.8%), and neomycin (7.1%). Neomycin had the highest prevalence among the included articles when compared to substances present in topical medications from the series. Promethazine was the second most positive active ingredient but showed significant variation among studies, with prevalence ranging from 0.8%^3^⁠⁠ to 5.9%,^2^ resulting in a lower average prevalence of positive tests per substance than neomycin, at 2.5%. Nitrofurazone and quinoline also had low average prevalence percentages in the tests of the included studies (2.7% and 2.6%, respectively).

The three least prevalent substances in all studies were triclosan (1%), p-tertiary butylphenol para-tertiary (0.9%), and anthraquinone (0.7%).

Regarding the analysis by age group, the most common allergens in children and adolescents, aged 1- to 19-years, considering a single study with n = 1253⁠⁠, were nickel sulfate (36.8%), thimerosal (18.4%), neomycin (6.4%), cobalt chloride, and fragrance mix (4.0% each), formaldehyde (3.2%), potassium dichromate and ethylenediamine (2.4% each). Children had positive reactions only to the nickel mix and fragrance mix.^3^

The systematic review also included an article presenting results in the elderly subpopulation (> 65-years)^8^: The main sensitizers in this age group were thimerosal (11%), neomycin (10.5%), nickel sulfate (10%), fragrance mix (10%), nitrofurazone (7%), potassium dichromate (6%), and paraphenylenediamine (5.5%).

### Associations between variables

Regarding the associations tested between the percentage of positive tests and the variables of sex, age, and region in Brazil, the results are described in Table 3. Values with p < 0.1 were considered statistically significant.

**Table 3:** Associations identified with sex, age, and region from the period of 2000 to 2022.

Blue: As the proportion of the variable increases, the proportion of positive tests decreases. Pink: As the proportion of the variable increases, the proportion of positive tests increases. Yellow: Statistically significant associations.

^a^ Southeast region considered as the reference.

#### Sex

A higher proportion of females in the sample was associated with a lower proportion of positive tests for the following substances: anthraquinone, potassium dichromate, carbamix, colophony, epoxy resin, hydroquinone, nitrofurazone, paraben mix, paraphenylenediamine, propylene glycol, quaternium 15, quinoline mix, and turpentine (p < 0.1).

For the substances ethylenediamine, formaldehyde, lanolin, mercapto mix, neomycin, nickel sulfate, thimerosal, and thiuram mix, a higher proportion of females in the sample was associated with a higher proportion of positive tests (p < 0.1).

#### Age

A higher average age in the study was associated with a lower proportion of positive tests for the substance’s potassium dichromate, carbamix, cobalt chloride, colophony, nitrofurazone, paraphenylenediamine, PPD mix, propylene glycol, p-tertiary butylphenol, and turpentine (p < 0.1).

Conversely, only for nickel sulfate, a higher average age in the study was associated with a higher proportion of positive tests (p < 0.1).

Brazilian regions of the studies: The substances formaldehyde, lanolin, quaternium 15, quinoline mix, and thimerosal appeared in a lower proportion of positive tests in the southern region, but without a statistically significant direct association (p > 0.1).

On the other hand, the substances anthraquinone, balsam of Peru, benzocaine, carbamix, colophony, epoxy resin, hydroquinone, triclosan, Kathon CG, paraben mix, perfume mix, paraphenylenediamine, PPD mix, propylene glycol, promethazine, quaternium 15, quinoline mix, p-tertiary butylphenol, and turpentine showed a higher proportion of positive tests in the studies conducted in the southeastern region (p < 0.1). The same suggestion is made for potassium dichromate, cobalt chloride, neomycin, nitrofurazone, and nickel sulfate, although no statistically significant significance was observed (p > 0.1).

No trends or associations were observed for the remaining substances.

### Variation in test positivity over time

The study observed an increasing trend in test positivity every 5-years for several substances, including Kathon CG, neomycin, and nickel sulfate also exhibited increasing test positivity (p < 0.1). Additionally, anthraquinone, benzocaine, potassium dichromate, balsam of Peru, cobalt chloride, colophony, formaldehyde, triclosan, p-tertiary butylphenol, PPD mix, paraphenylenediamine, perfume mix, propylene glycol, and turpentine (p > 0.1).

Conversely, there was a decreasing test positivity trend, every 5-years, for quaternium 15 and thimerosal (p < 0.1), epoxy resin, ethylenediamine, hydroquinone, lanolin, mercapto mix, nitrofurazone, paraben mix, promethazine, thiuram mix, and quinoline mix (p > 0.1). The results are described in Table 4.

**Table 4:** Positivity variation of patch test results over time, using the Brazilian Standard Battery, between 2000‒2022 (n = 4,703).

Substance	-| 1999^a^	2000‒2005	2005‒2010	2011 |-	ß (pp)^b^
Antraquinone	0.5	0.3	1.4	0.6	0.07
Benzocaine	0.5	0.9	1.3	1.7	0.28
Potassium Dichromate	8.1	6.0	8.0	9.6	0.73
Peru Balsam	3.2	2.8	3.4	4.9	0.54
Carba mix	3.7	3.4	5.4	5.0	0.42
Cobalt Chloride	11.0	8.6	10.4	13.0	0.91
Colophony	2.6	1.8	3.1	4.7	0.71
Epoxy Resin	1.8	2.8	2.0	2.0	-0.07
Ethylenediamine	3.1	3.4	3.9	2.9	-0.11
Formaldehyde	3.8	4.5	4.6	4.7	0.14
Hydroquinone	1.3	1.0	2.9	1.0	-0.04
Triclosan	0.7	0.1	1.6	1.5	0.31
Kathon CG	2.2	0.0	3.7	15.2	4.02
Lanolin	1.1	2.7	1.2	0.6	-0.39
Mercapto mix	3.7	1.5	1.8	1.2	-0.41
Neomycin	4.3	9.3	8.2	11.7	1.41
Nitrofurazone	3.7	3.4	1.9	1.4	-0.61
P-Tertiary Butylphenol	0.7	1.0	1.1	3.4	0.74
PPD mix	1.8	3.7	6.2	4.3	0.44
Paraben mix	2.7	2.0	2.6	1.6	-0.21
Paraphenylenediamine	8.7	6.2	7.7	9.2	0.51
Perfume mix	8.4	7.3	6.4	12.1	1.22
Promethazine	1.8	2.0	3.5	0.4	-0.43
Propylene Glycol	0.8	0.6	1.3	1.3	0.17
Quaternium 15	11.3	4.4	1.6	1.4	-1.85
Quinoline mix	6.0	2.3	1.6	1.4	-0.78
Nickel Sulfate	25.1	36.2	33.3	44.9	4.06
Turpentine	1.4	0.0	2.4	0.8	0.03
Thimerosal	16.3	18.4	16.6	10.4	-1.98
Thiuram mix	5.5	5.4	3.6	3.7	-0.49

a Articles published between 2000‒2022 were included. Data collection in two included articles was conducted between 1995‒2000.

b Linear regression coefficient (in percentage points). Positive results = average increase; Negative results = average decrease.

## Discussion

The primary objective of this study was to compile and compare the results of patch tests conducted on the Brazilian population, specifically focusing on patients suspected of contact dermatitis, from 2000 to 2022.

Consistent with global literature, nickel was identified as the main sensitizing agent across all studies. The positivity rates found in the Brazilian population in this study (31.8%) were higher compared to more recent reports in the European population, where nickel sensitization was found in approximately 13%‒17% of adults, around 10% of adolescents, and 7%‒9% of children.^14^⁠⁠

Metals emerged as significant sensitizers in most studies and are among the primary allergens causing contact dermatitis. Apart from nickel, cobalt and chromium were notable sensitizers, likely due to the high prevalence of metal-containing products to which the Brazilian population is exposed, such as jewelry, piercings, cleaning products, eyewear, watches, buttons on clothing, and gold-plated items, along with the presence of metals in occupational dermatoses.^15^⁠

According to Spiewak et al.,^14^ the most common sensitizers in the European population, in addition to nickel sulfate, are thimerosal and fragrances. Similarly, in the systematic review, thimerosal ranked among the top 10 most frequent sensitizers in all articles. The authors emphasize its presence in medications, vaccine preservatives, contact lens solutions, and tattoo inks, which may contribute to the high prevalence of sensitization to this substance⁠⁠.^2^⁠

Regarding substances with a lower prevalence of positive reactions in patch tests, this study identified triclosan, p-tert-butylphenol, and anthraquinone, with average prevalence percentages of up to 1%. Considering the very low frequency of positive tests for these substances, the authors can question their real importance in the etiology of allergic contact dermatitis in the Brazilian population over the past two decades and the need to include them in the standard Brazilian patch test panel.

These results align with published data reporting infrequent contact sensitization to triclosan. The Swiss Contact Dermatitis Research Group reported a triclosan sensitization prevalence of 0.8% in a large study involving 2,295 patients.^16^⁠ Schena et al.^17^⁠ confirmed a low incidence of triclosan sensitization, mainly associated with the use of deodorants containing this substance in their study.

Due to reported potential risks ranging from allergies to endocrine disruption, triclosan was banned by the FDA in September 2016 in soaps (liquid, gel, foam, bar), while the European Union (EU) prohibited its use in all biocidal products for human hygiene starting in January 2017, which may explain the reduction in positive tests for triclosan.^18^⁠

A study by Fransway et al.^19^⁠ from the North American Contact Dermatitis Group in 2013 also reported a decreased prevalence of positive reactions to p-tert-butylphenol, a substance commonly used in adhesives, including shoe production, among other substances, when compared to rates observed in the previous ten years.

Regarding anthraquinones, they have industrial applications in the production of hydrogen peroxide, paints, and medications such as laxatives and cathartics. They are also present in plants like Senna, Cascara sagrada, Rhubarb, and Aloe⁠.^20^, ^21^⁠ Anthraquinone is not included in the standard American and European patch test panels, with limited published data on its prevalence of positive reactions in patch tests.

A tendency towards a higher proportion of positive tests in women was found for a number of substances, possibly related to the greater frequency of exposure occurring in this group. Among these are ethylenediamine, primarily used in topical medications such as antifungals, antibacterial, and corticosteroids; formaldehyde, utilized in the formulation of cosmetics, personal hygiene products, and nail polishes; lanolin, a cutaneous emollient used in the cosmetics industry; mercapto mix, employed in the rubber industry and found in gloves, condoms, toys, shoes, among other items; thimerosal, utilized as a preservative in vaccines; thiuram mix, used in the rubber industry for fungicides and pesticides, as well as repellents; neomycin, an over-the-counter antibacterial medication in Brazil; and nickel sulfate, found in jewelry, earrings, watches, bracelets, denim buttons, needles, zippers, coins, keys, buckles, eyeglass frames, orthodontic and orthopedic appliances, foods, among others.

Additionally, the authors found a higher tendency of positive test proportions for a multitude of substances in the southeastern region compared to the southern region (anthraquinone, balsam of Peru, benzocaine, carbamix, colophony, epoxy resin, hydroquinone, triclosan, Kathon CG, paraben mix, perfume mix, paraphenylenediamine, PPD mix, propylene glycol, promethazine, quaternium 15, quinoline mix, p-tertiary butylphenol, and turpentine). This finding may be justified by the greater number of studies conducted in the southeastern region (7 out of 10 studies) included in the analysis.

Moreover, a higher average age in the study population, encompassing children, adults, and the elderly, was associated with a lower proportion of positive tests for substances such as potassium dichromate, carbamix, cobalt chloride, colophony, nitrofurazone, paraphenylenediamine, PPD mix, propylene glycol, p-tertiary butylphenol, and turpentine. A possible explanation lies in the association of these substances with occupational activities such as construction work and industries like rubber, leather, and metals, which tend to be less common in older age groups.

The article by Duarte et al.^11^⁠ evaluated the variation in positivity rates for substances included in the standard Brazilian patch test panel recommended by the Brazilian Group of Contact Dermatitis Studies between 2006 and 2011. Three substances showed a statistically significant decrease in sensitization rates over the studied years: lanolin (p = 0.01), neomycin (p = 0.01), and anthraquinone (p = 0.04). The systematic review, with more robust strength, also found a decreasing percentage, every five years on average, for lanolin; however, it observed an increasing percentage for neomycin and anthraquinone. Historically, exposure to certain allergens shows variation according to geographical areas, such as neomycin, which remains more common in North America than in Europe.^22^⁠ The overall prevalence of neomycin sensitization has decreased in several countries in recent years, possibly due to its restricted availability in some locations⁠.^23^⁠ In Brazil, neomycin is still present in over-the-counter topical medications with high consumption by the Brazilian population, which may explain the observed increase in percentage in this study.

The findings of this review regarding the frequency of common allergens in the pediatric and adolescent population are consistent with global literature.^10^ However, the present study was limited by the small number of articles including this age group. Nickel sulfate was the most frequently positive allergen in patch tests conducted on the pediatric population in Brazil, which aligns with a previous systematic review⁠.^24^⁠ In the same review, lanolin was considered one of the top 10 most frequently positive substances, unlike the results of this study, where the authors did not observe any positive reactions to this allergen in the pediatric and adolescent population.

Despite the rigorous methodology employed in this systematic review, several limitations must be acknowledged. One significant limitation is the small number of studies included in the analysis. Eight articles had to be excluded after initial eligibility, according to exclusion criteria described in the methodology: six for not presenting patch test results^25^, ^26^, ^27^, ^28^, ^29^, ^30^ and two for partially presenting patch test results.^31^, ^32^ The limited number of eligible articles restricts the generalizability of the findings. This scarcity of studies likely reflects the early stage of research in this domain within Brazil, pointing to opportunities for further investigation and expansion.

In the most recent review of the relevant literature, the authors found, after 2022, only one article that would be possible to integrate into this systematic review, which was carried out retrospectively to investigate hand contact dermatitis. This study found in a total of 173 patients, 61.8% of irritant dermatitis and only 23.1% of allergic dermatitis and 5.2% of atopic dermatitis. The most frequent allergens were kathon cg (42%), nickel sulfate (33%) and thiuram-mix 18%.^33^ The profile is compatible with the data observed in the 2000‒2022 historical series current in this systematic review.

Another critical limitation pertains to the overall quality of the included studies. While the majority of the selected articles were of average methodological quality, which may increase the risk of bias, it is noteworthy that high-quality studies were also included, and no low-quality studies were admitted into the review.

## Conclusion

The most frequently identified allergens in patch tests in this SR were nickel sulfate, thimerosal and cobalt. Triclosan, tertiary P-butyphenol and anthraquinone were the three least prevalent substances. A trend has been demonstrated for test positivity to increase every 5-years on average for kathon CG, neomycin, and nickel sulfate.

This systematic review offers valuable insights, despite some limitations. Although the number of included studies is small, this highlights the potential for further exploration in this research area. The compilation of these data will enable healthcare professionals working with dermatological allergies to gain a more concrete understanding of the prevalence of positive contact test reactions to each substance present in the Brazilian standard battery. These findings, in addition to their clinical relevance, may serve as a foundation for recommending useful and appropriate series of contact tests, allowing for a more comprehensive and accurate evaluation of patients suspected of allergic contact dermatitis in Brazil in the future.

## Financial support

None declared.

## Authors’ contributions

Ana Laura Andrade Bueno: Study conception and planning; data collection, analysis and interpretation; statistical analysis; intellectual participation in propaedeutic and/or therapeutic management of studied cases; preparation and writing of the manuscript; effective participation in research orientation; manuscript critical review; approval of the final version of the manuscript.

Nathalia Hoffmann Guarda Aguzzoli: Study conception and planning; data collection, analysis and interpretation; statistical analysis; intellectual participation in propaedeutic and/or therapeutic management of studied cases; approval of the final version of the manuscript.

Renan Rangel Bonamigo: Study conception and planning; data collection, analysis and interpretation; statistical analysis; intellectual participation in propaedeutic and/or therapeutic management of studied cases; preparation and writing of the manuscript; effective participation in research orientation; critical literature review; approval of the final version of the manuscript.

## Conflicts of interest

None declared.
